# Escitalopram Targets Oxidative Stress, Caspase-3, BDNF and MeCP2 in the Hippocampus and Frontal Cortex of a Rat Model of Depression Induced by Chronic Unpredictable Mild Stress

**DOI:** 10.3390/ijms22147483

**Published:** 2021-07-13

**Authors:** Vlad Dionisie, Adela Magdalena Ciobanu, Vlad Alexandru Toma, Mihnea Costin Manea, Ioana Baldea, Diana Olteanu, Alexandra Sevastre-Berghian, Simona Clichici, Mirela Manea, Sorin Riga, Gabriela Adriana Filip

**Affiliations:** 1 Department of Psychiatry and Psychology, ‘Carol Davila’ University of Medicine and Pharmacy, 020021 Bucharest, Romania; vlad.dionisie@gmail.com (V.D.); mirelamanea2003@yahoo.com (M.M.); 2 Department of Psychiatry, ‘Prof. Dr. Alexandru Obregia’ Clinical Hospital of Psychiatry, 041914 Bucharest, Romania; adela.ciobanu@yahoo.com; 3 Neuroscience Department, Discipline of Psychiatry, ‘Carol Davila’ University of Medicine and Pharmacy, 020021 Bucharest, Romania; 4 Department of Molecular Biology and Biotechnology, Faculty of Biology and Geology, Babes-Bolyai University, 400028 Cluj-Napoca, Romania; 5 Department of Biochemistry and Experimental Biology, Institute of Biological Research, Branch of NIRDBS Bucharest, 400113 Cluj-Napoca, Romania; 6 Department of Molecular and Biomolecular Physics, NIRD for Isotopic and Molecular Technologies, 400293 Cluj-Napoca, Romania; 7 Department of Physiology, “Iuliu Hatieganu” University of Medicine and Pharmacy, 400006 Cluj-Napoca, Romania; baldeaioana@gmail.com (I.B.); ariana_di@yahoo.com (D.O.); alexandra_berghian@yahoo.com (A.S.-B.); simonaclichici@yahoo.com (S.C.); adrianafilip33@yahoo.com (G.A.F.); 8 Department of Stress Research and Prophylaxis, ‘Prof. Dr. Alexandru Obregia’ Clinical Hospital of Psychiatry, 041914 Bucharest, Romania; D_S_Riga@yahoo.com; 9 Romanian Academy of Medical Sciences, 927180 Bucharest, Romania

**Keywords:** escitalopram, antidepressant, chronic unpredictable mild stress, depression, caspase, oxidative stress, antioxidant, brain derived neurotrophic factor, MeCP2, oligodendrocyte

## Abstract

In recent years, escitalopram (ESC) has been suggested to have different mechanisms of action beyond its well known selective serotonin reuptake inhibition. The aim of this study is to investigate the effects of escitalopram on oxidative stress, apoptosis, brain-derived neurotrophic factor (BDNF), Methyl-CpG-binding protein 2 (MeCP2), and oligodendrocytes number in the brain of chronic unpredictable mild stress-induced depressed rats. The animals were randomised in four groups (8 in each group): control, stress, stress + ESC 5 and stress + ESC 5/10. ESC was administered for 42 days in a fixed dose (5 mg/kg b.w.) or in an up-titration regimen (21 days ESC 5 mg/kg b.w. then 21 days ESC 10 mg/kg b.w.). Sucrose preference test (SPT) and elevated plus maze (EPM) were also performed. ESC improved the percentage of sucrose preference, locomotion and anxiety. ESC5/10 reduced the oxidative damage in the hippocampus and improved the antioxidant defence in the hippocampus and frontal lobe. ESC5/10 lowered caspase 3 activity in the hippocampus. Escitalopram had a modulatory effect on BDNF and the number of oligodendrocytes in the hippocampus and frontal lobe and also improved the MeCP2 expressions. The results confirm the multiple pathways implicated in the pathogenesis of depression and suggest that escitalopram exerts an antidepressant effect via different intricate mechanisms.

## 1. Introduction

Depression is one of the most common psychiatric disorders, with a global prevalence and an annual incidence estimated to be 4.4% and 3%, respectively [1,2]. World Health Organization predicted that beginning with 2030 depression will be the illness with the highest burden of disease [1]; 10–30% of people with depression are treatment resistant and have a low quality of life and functional impairments [3].

Unfortunately, despite major progress in the research of this disorder, the exact pathogenic mechanisms involved in the occurrence of depression and their maze of interactions are still under debate. Mounting evidence suggests the implication of oxidative stress in the pathomechanism of depression. Oxidative stress is viewed as the imbalance between the reactive oxygen species (ROS) (i.e., free radicals and other reactive metabolites), and the level of antioxidant molecules [4]. Numerous studies have reported alterations in the antioxidant defense (i.e., glutathione peroxidase-GPx, glutathione reduced-GSH, vitamin E) and increased oxidative markers (i.e., catalase activity-CAT) in patients with depression, although conflicting results are reported in the literature. Despite contradictory data, the implication of oxidative stress is attested by a large body of evidence [5,6].

It has been shown that there is a link between psychological stress and depression, meaning that stressful life events contribute to the onset of major depression and that stress experienced during childhood is a risk factor for depression later in life [7,8,9]. In animal models, external stressors determine behavioural signs that parallel the symptoms seen in depressive patients, including anhedonic and anxiety behaviours [10]. External stressors determine the activation of the hypothalamic-pituitary-adrenal (HPA) axis. Particularly, if stress becomes chronic, it determines the dysregulation of the HPA axis and consequently an abnormal and sustained increase of glucocorticoids levels, which leads to oxidative stress [11,12,13,14]. In addition, chronic stress is a source of ROS through the activation of brain microglia or NF-kB pathway that in turn will produce pro-inflammatory cytokines [15]. Oxidative stress and inflammation have an interdependent relationship and their upregulation is stimulated bidirectionally via different mechanisms (e.g., NF-kB pathways) leading in the end to a vicious circle [16]. Increased reactive oxygen species produce mitochondrial dysfunction and damages to the DNA and membrane lipids, finally leading to cell apoptosis [17].

Moreover, in animal studies, chronic stress has been shown to promote neuronal apoptosis also through upregulation of caspases activity via increased caspase-3 activity or Bcl-2 reduction and downregulation of neurotrophic factors such as brain-derived neurotrophic factor (BDNF). Chronic mild stressed mice showed increased caspase-3 and Bax positive cells in the hippocampus as compared to control [18]. These effects decreased neurogenesis and produced neuronal cell damage and apoptosis along with the occurrence of depressive behaviour ultimately proving the pro-apoptotic and anti-neurogenic effect of chronic stress [13,14,19].

BDNF is an important neurotrophin involved in the neuronal survival, differentiation, and development [7,15]. Depressive disorder is associated with marked reduced levels of BDNF, both in humans and different animal models including chronic unpredictable mild stress (CUMS) paradigm. Depression is also accompanied by altered hippocampal or frontal brain structures and it has been consistently suggested that this might be a consequence of lowered BDNF [20,21,22,23,24]. In addition, BDNF knockdown rats showed depressive-like behaviour and had reduced neurogenesis in the hippocampus [25]. Also, BDNF is thought to be implicated in the mechanism of action of selective serotonin reuptake inhibitors since remission of symptoms after treatment is associated with restored BDNF levels [23,26,27]. Recent rodent studies indicated that BDNF might have a role in preventing the increase in ROS found in depression most probably via a nuclear factor erythroid-derived 2-like 2 (Nrf2) dependent mechanism [11,28]. Nrf2 is a transcription factor induced by oxidative stress and orchestrates the promotion of antioxidant enzymes (such as catalase or GPx) synthesis. Nrf2 was found to be decreased in humans or rodent stress models of depression along with decreased levels of antioxidant defense [29,30,31]. More precisely, Martin-Hernandez et al. (2018) documented a decrease in the Nrf2 expression in the dorsolateral prefrontal cortex of patients with major depressive disorder [32]. Also, mice subjected to a social defeat stress paradigm showed decreased levels of Nrf2 protein expression in the hippocampus (i.e., CA3 and dentate gyrus) and the prefrontal cortex [33]. Nrf2 knock out mice subjected to chronic stress showed a depressive phenotype and lower antioxidant capacity which could be prevented by antioxidant administration [30].

Methyl-CpG-binding protein 2 (MeCP2) is involved in the epigenetic regulation. In the brain, one of the key regulators of BDNF expression is MeCP2. Classically, the loss of MeCP2 function by mutation is involved in the pathogenesis of Rett syndrome [34]. Interestingly, recent research has demonstrated that MeCP2 is involved in the development of chronic-stress-induced depressive behaviour and that expression of MeCP2 is decreased in the hippocampus of depressed rats [35,36]. Also, it was demonstrated that signalling pathways activated by monoamine neurotransmitters via antidepressants target MeCP2. Hutchinson et al. (2012) showed that mice bearing a genetic knock-in mutation that eliminates the phosphorylation site of MeCP2 exhibited depressive-like behaviours and also did not respond to chronic treatment with the antidepressant imipramine [36]. Moreover, current evidence suggest that MeCP2 could have some protective effect against oxidative damage, since the most recommended treatments target oxidative stress. Filosa et al. (2015) proposed that, based on its role in chromatin dynamics, MeCP2 could interfere with genes involved in antioxidant and radical scavengers mechanisms [37].

Therefore, in this study, our aim is to evaluate, in a CUMS rat model of depression, the underlying mechanisms involved in the antidepressant effect of escitalopram (ESC), a selective serotonin reuptake inhibitor. More precisely, we aimed to assess whether the antidepressant effects of escitalopram would be related to the regulation of oxidative stress, BDNF and MeCP2 levels in the hippocampus and frontal cortex. Also, in parallel, the histopathological examination of the hippocampus and frontal lobe as well as the behavioural testing (elevated plus maze–EPM, sucrose preference test–SPT) of animals were performed.

## 2. Results

### 2.1. The Effect of CUMS Procedure and Escitalopram Treatment on Sucrose Preference

**Figure 1:**
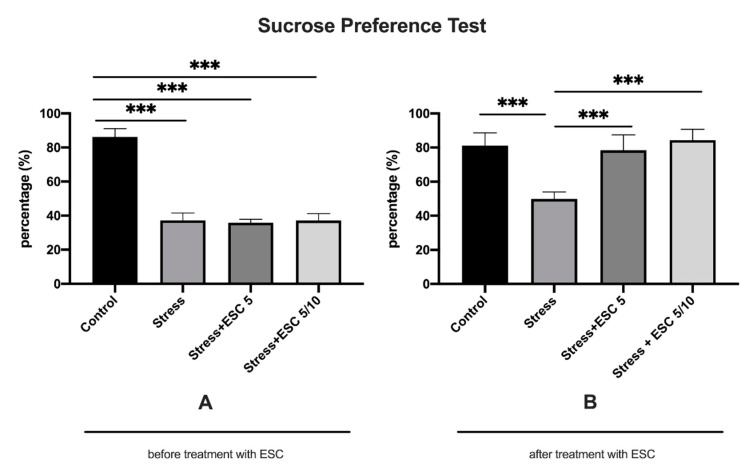
The influence of CUMS and escitalopram treatment on the percentage of sucrose preference. After 21 days of CUMS rats displayed a decreased percentage of sucrose preference as compared to controls (**A**). After 6 weeks of antidepressant treatment, the groups stress + ESC5 and stress + ESC5/10 had significantly improved sucrose preference percentage in comparison with the stress group (**B**) (*p* < 0.001). Each group consisted of 8 rats; ESC5 group received (oral route) escitalopram 5 mg/kg b.w. for 42 days; ESC 5/10 group received (oral route) 5 mg/day b.w. escitalopram for 21 days then 10 mg/kg b.w. escitalopram (oral route) for 21 days; results are expressed as mean ± SD; ANOVA and Tukey’s *post hoc* test; *** *p* < 0.001; CUMS, chronic unpredictable mild stress; ESC, escitalopram.

After 3 weeks of chronic unpredictable mild stress, animals had a percentage of sucrose preference under 65%, which indicated depressive-like behaviour. Control animals had an increased sucrose preference percentage as compared to stress, stress + ESC 5 and stress + ESC 5/10 groups (86.19 ± 4.88 vs. 37.19 ± 4.31, *p* < 0.001; 86.19 ± 4.88 vs. 35.92 ± 1.96, *p* < 0.001; 86.19 ± 4.88 vs. 37.14 ± 4.10, *p* < 0.001) (Figure 1A). After 6 weeks of escitalopram treatment, chronic stress maintained a significant decrease in the percentage of sucrose preference as compared to the control group in the stress exposed group (81.13 ± 7.49 vs. 49.84 ± 4.09, *p* < 0.001). Escitalopram treatment, either the 5 mg/kg b.w. dose or the up-titration to 10 mg/kg b.w regimen, determined an antidepressant effect and significantly increased the sucrose preference percentage in comparison to the stress group (78.44 ± 9.04 vs. 49.84 ± 4.09, *p* < 0.001 and 84.39 ± 6.34 vs. 49.84 ± 4.09, *p* < 0.001, respectively) (Figure 1B).

### 2.2. EPM Evaluation

Even though EPM is a widely employed animal behavioural model of anxiety, total and closed arms travelled distance and total and closed arms entries assess general locomotion in EPM.

**Figure 2:**
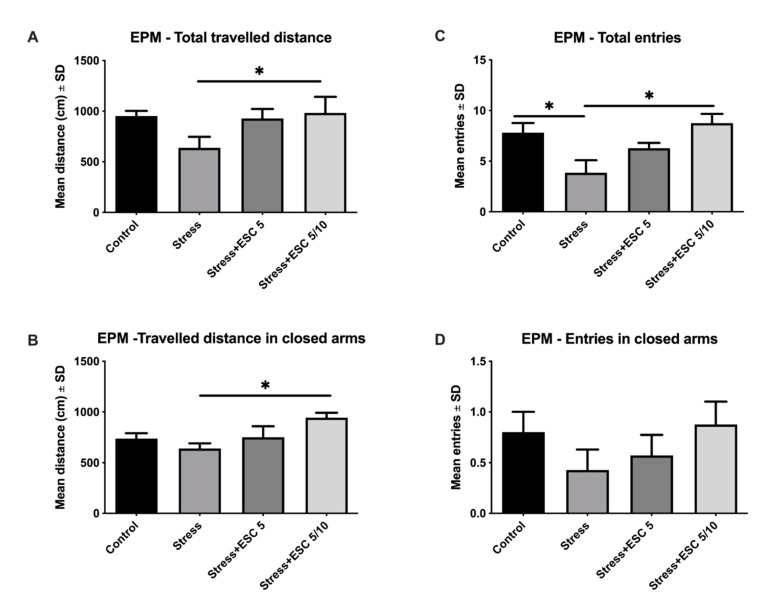
The effects of chronic unpredictable mild stress and escitalopram treatment on the total travelled distance (**A**), travelled distance in closed arms (**B**), total entries (**C**) and entries in closed arms (**D**) in the elevated plus maze. In elevated plus maze, Stress + ESC5/10 group, in comparison to stress group, exhibited significantly higher locomotor activity (total travelled distance (**A**), travelled distance in closed arms (**B**) and total number of entries (**C**) (*p* < 0.05). Each group consisted of 8 rats; ESC5 group received (oral route) escitalopram 5 mg/kg b.w. for 42 days; ESC 5/10 group received (oral route) 5 mg/day b.w. escitalopram for 21 days then 10 mg/kg b.w. escitalopram (oral route) for 21 days. Results are expressed as mean ± SD; ANOVA and Dunn’s *post hoc* test; * *p* < 0.05; ESC, escitalopram.

Regarding locomotion, CUMS reduced the number of total entries as compared to control group (*p* < 0.05) (Figure 2C) and tended to decrease (1.49 times) the total travelled distance (Figure 2A), travelled distance (1.15 times) and entries (1.86 times) in closed arms (Figure 2B,D) as compared to the control group, but without any statistical significance (*p* > 0.05). ESC 5/10 treatment improved locomotion and increased the total travelled distance, total entries and travelled distance in closed arms in comparison with the stress group (*p* < 0.05) (Figure 2A–C). ESC5/10 also increased (2.04 times) the number of entries in closed arms, but without statistical significance (*p* > 0.05) (Figure 2D).

**Figure 3:**
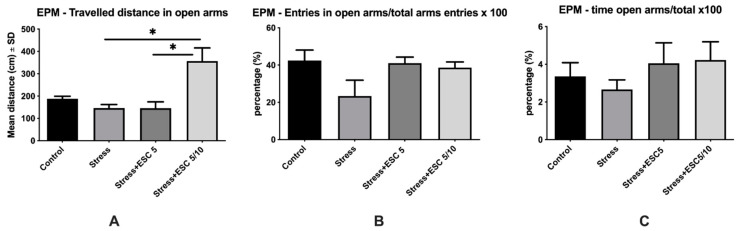
The effects of chronic unpredictable mild stress and escitalopram treatment on emotionality in elevated plus maze (EPM). The ESC5/10 treated rats travelled significantly greater distance both as compared to ESC5 and stress group (*p* < 0.05) (**A**). Stress + ESC5 and Stress + ESC5/10 groups had higher percentages of entries in open arms (**B**) and time spent in open arms (**C**) than the stress group, but without statistical significance (*p* > 0.05). Each group consisted of 8 rats; ESC5 group received (oral route) escitalopram 5 mg/kg b.w. for 42 days; ESC 5/10 group received (oral route) 5 mg/day b.w. escitalopram for 21 days then 10 mg/kg b.w. escitalopram (oral route) for 21 days. Results are expressed as mean ± SD; ANOVA and Dunn’s *post hoc* test; * *p* < 0.05; ESC, escitalopram.

Generally, in the EPM, the percentage of entries and the travelled distance in the open arms, as well as the percentage of time spent in open arm from the total time quantify the anxiety. Thus, CUMS increased the anxiety and decreased the percentage of entries in the open arms from total arm entries in comparison with the control group, but with differences without statistical significance (*p* > 0.05) (Figure 3B). Also, stress induction tended to decrease (1.28 times) the travelled distance in open arms (Figure 3A) and the percentage of time spent in open arm from total time (Figure 3C) (1.26 times) as compared to the control group, but without statistical significance (*p* > 0.05). ESC 5/10 group improved the travelled distance in open arms, which means lower anxiety level, in comparison with the depressed animals (*p* < 0.05) (Figure 3A). The up-titration escitalopram administration (ESC5/10) slightly improved the percentage of time spent in the open arms of the EPM (1.58 times) and the percentage of entries in open arms (1.65 times), but without statistical significance (*p* > 0.05) (Figure 3B,C).

### 2.3. Oxidative Stress Assessment in the Hippocampus and Frontal Cortex

**Figure 4:**
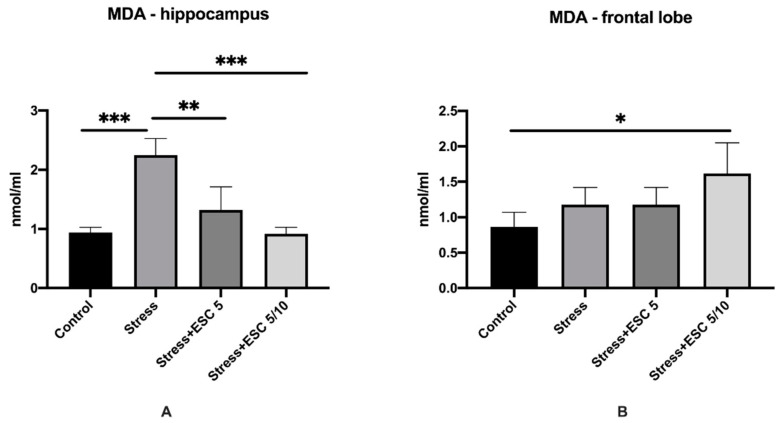
The effects of CUMS and escitalopram treatment in different regimens on the levels of MDA in the hippocampus (**A**) and frontal lobe (**B**) of adult rats. MDA displayed higher values in the hippocampus of stress group (**A**) (*p* < 0.001). Both escitalopram dose regimens (ESC 5 and ESC5/10) decreased the MDA levels in the hippocampus of stressed rats (*p* < 0.01 and *p* < 0.001, respectively) (**A**). MDA was increased in the frontal lobe of stress + ESC 5/10 group (*p* < 0.05) (**B**). Each group consisted of 8 rats; ESC5 group received (oral route) escitalopram 5 mg/kg b.w. for 42 days; ESC 5/10 group received (oral route) 5 mg/day b.w. escitalopram for 21 days then 10 mg/kg b.w. escitalopram (oral route) for 21 days. Results are expressed as mean ± SD; ANOVA and Tukey’s *post hoc* test; * *p* < 0.05; ** *p* < 0.01; *** *p* < 0.001; CUMS, chronic unpredictable mild stress, ESC, escitalopram; MDA, malondialdehyde.

Malondialdehyde (MDA) levels in the hippocampus and frontal cortex are illustrated in Figure 4. The animals exposed only to CUMS had higher levels of MDA in the hippocampus compared to non-stressed rats (2.24 ± 0.28 vs. 0.93 ± 0.08, *p* < 0.001). MDA levels in the hippocampus decreased significantly in the Stress + ESC 5/10 and Stress + ESC 5 groups as compared with the Stress group (0.91 ± 0.11 vs. 2.24 ± 0.28, *p* < 0.001 and 1.32 ± 0.39 vs. 2.24 ± 0.28, *p* < 0.01 respectively) (Figure 4A). In the frontal lobe, MDA displayed higher levels in the stress + ESC 5/10 versus control (1.61 ± 0.43 vs. 0.86 ± 0.20, *p* < 0.05). The stress group had also increased MDA in the frontal cortex in comparison with controls, but the differences did not reach statistical significance (1.17 ± 0.24 vs. 0.86 ± 0.20, *p* > 0.05) (Figure 4B).

**Figure 5:**
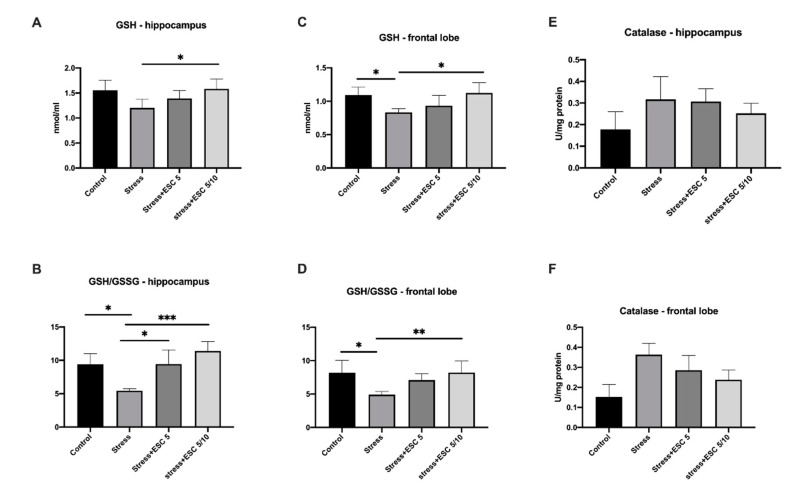
The effects of CUMS and escitalopram treatment on the antioxidant defence (GSH, GSH/GSSG, catalase). CUMS increased decreased GSH and GSH/GSSH levels in the frontal lobe (*p* < 0.05) (**C**) and hippocampus and frontal lobe respectively (*p* < 0.05 and *p* < 0.05, respectively) (**B**,**D**). Escitalopram dose-increment treatment increased the GSH levels in the hippocampus (*p* < 0.05) (**A**) and frontal lobe (*p* < 0.05) (**B**) and the GSH/GSSG levels in the hippocampus (*p* < 0.001) (**B**) and frontal lobe (*p* < 0.01) (**D**). Escitalopram administration for 42 days increased the GSH/GSSG levels in the hippocampus (*p* < 0.05) (**B**). CUMS increased the catalase levels in the hippocampus (**E**) and frontal lobe (**F**), but without statistical significance (*p* > 0.05). Stress + ESC 5/10 group had lower levels of catalase in the hippocampus (**E**) and frontal lobe (**F**) as compared to the Stress group, but the differences did not reach statistical significance (*p* > 0.05). Each group consisted of 8 rats; ESC5 group received (oral route) escitalopram 5 mg/kg b.w. for 42 days; ESC 5/10 group received (oral route) 5 mg/day b.w. escitalopram for 21 days then 10 mg/kg b.w. escitalopram (oral route) for 21 days. Results are expressed as mean ± SD; ANOVA and Tukey’s *post hoc* test; * *p* < 0.05; ** *p* < 0.01; *** *p* < 0.001; CUMS, chronic unpredictable mild stress; GSH, glutathione reduced; GSSG, glutathione oxidised; ESC, escitalopram.

Escitalopram administered in the up-titration regimen increased GSH levels in both hippocampus and frontal lobe as compared to rats subjected only to chronic stress (1.58 ± 0.19 vs. 1.20 ± 0.17, *p* < 0.05 and 1.12 ± 0.15 vs. 0.83 ± 0.05, *p* < 0.05 respectively). In addition, GSH levels, in the frontal cortex, were decreased in the stress group animals versus controls (0.83 ± 0.05 vs. 1.09 ± 0.11, *p* < 0.05) (Figure 5A,C).

The glutathione reduced/glutathione oxidised (GSH/GSSG) ratios were significantly improved in the hippocampus and frontal lobe of rats from the Stress + ESC5/10 group versus stress group (11.41 ± 1.42 vs. 5.45 ± 0.27, *p* < 0.001 and 8.22 ± 1.72 vs. 4.90 ± 0.47, *p* < 0.01, respectively). Moreover, the control group had increased GSH/GSSG ratio in comparison with the stress group, both in the hippocampus and frontal lobe (9.396 ± 1.61 vs. 5.45 ± 0.27, *p* < 0.05 and 8.18 ± 1.87 vs. 4.90 ± 0.47, *p* < 0.05, respectively) (Figure 5B,D).

Regarding catalase, the activity of the enzyme in the hippocampus and frontal lobe did not change significantly in the groups (*p* > 0.05) (Figure 5E,F).

### 2.4. Caspase-3 Activity in the Hippocampus and Frontal Cortex

**Figure 6:**
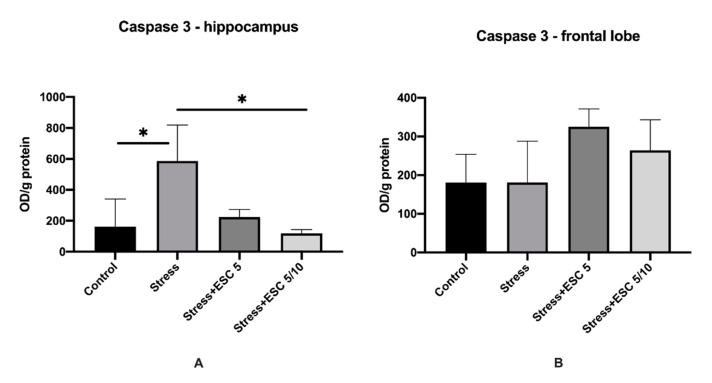
The effects of CUMS and escitalopram on the caspase-3 activity. CUMS regimen increased the activity of caspase 3 in the hippocampus of stress group (*p* < 0.05), (**A**). ESC 5/10 group had a decreased levels of activity of caspase 3 in the hippocampus in comparison with the stress group (*p* < 0.05) (**A**). Stress + ESC 5 and Stress+ ESC5/10 groups had higher levels of caspase-3 in the frontal lobe (**B**), but without statistical significance (*p* > 0.05). Each group consisted of 8 rats; ESC5 group received (oral route) escitalopram 5 mg/kg b.w. for 42 days; ESC 5/10 group received (oral route) 5 mg/day b.w. escitalopram for 21 days then 10 mg/kg b.w. escitalopram (oral route) for 21 days. Results are expressed as mean ± SD; ANOVA and Tukey’s *post hoc* test; * *p* < 0.05; CUMS, chronic unpredictable mild stress, ESC, escitalopram.

In the hippocampus, animals from the Stress + ESC5/10 group showed decreased levels of caspase-3 when compared to the stress group (119.1 ± 23.54 vs. 586.3 ± 232.5, *p* < 0.05). In the frontal cortex, caspase-3 levels were higher in the group treated with the up-titration regimen of escitalopram or with a fixed dose of 5 mg/day escitalopram versus the stress group, but the differences were not statistically significant (264.0 ± 79.22 vs. 184.1 ± 106.5, *p* > 0.05 and 325.2 ± 45.83 vs. 184.1 ± 106.5, *p* > 0.05) (Figure 6A,B).

### 2.5. Quantitative Estimation of BDNF and MeCP2 Expressions

**Figure 7:**
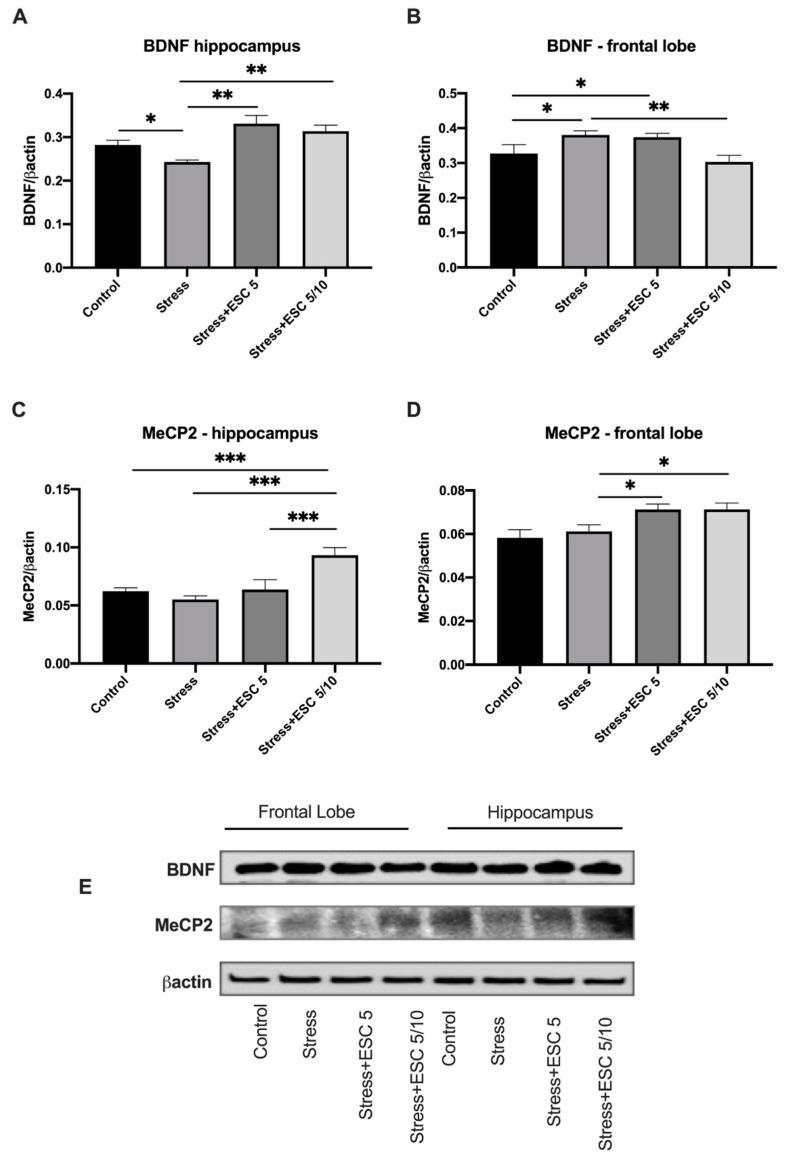
The effects of CUMS and escitalopram administration on the expression of BDNF and MeCP2 in the brain. Expression of BDNF and MeCP2 were analysed by western blot. The results were normalised to βactin. Stress reduced BDNF levels in the hippocampus (**A**) while escitalopram administration in both regiments increased the levels of BDNF in the hippocampus. Stress increased the BDNF levels in the frontal lobe (**B**) and escitalopram up-titration regiment normalised the levels of BDNF (**B**). Stress + ESC 5/10 had increased levels of MeCP2 levels in the hippocampus (**C**) and in the frontal lobe (**D**). (**E**) Representative western blot images. Each group consisted of eight rats; ESC5 group received (oral route) escitalopram 5 mg/kg b.w. for 42 days; ESC 5/10 group received (oral route) 5 mg/day b.w. escitalopram for 21 days then 10 mg/kg b.w. escitalopram (oral route) for 21 days; Results are expressed as mean ± SD; ANOVA and Tukey’s *post hoc* test; * *p* < 0.05; ** *p* < 0.01; *** *p* < 0.001; CUMS, chronic unpredictable mild stress, ESC, escitalopram, BDNF, brain-derived neurotrophic factor, MeCP2, Methyl-CpG-binding protein 2.

BDNF in the hippocampus of rats from stress + ESC5 or stress + ESC5/10 group increased compared to the levels of stressed animals (*p* < 0.01 and *p* < 0.01, respectively) (Figure 7A). Conversely, ESC5/10 decreased the BDNF expressions in the frontal lobe compared to the stress untreated group (*p* < 0.01). BDNF levels in frontal cortex of untreated depressed rats were higher than in controls (*p* < 0.05), while in the hippocampus BDNF decreased in the stress group versus control group (*p* < 0.05) (Figure 7A,B).

In the hippocampus and frontal cortex, MeCP2 expressions increased in the group exposed to stress and treated with ESC 5/10 (*p* < 0.001 and *p* < 0.05, respectively) (Figure 7C,D). Moreover, the stress + ESC5 group had increased MeCP2 levels in the frontal cortex (*p* < 0.05). Chronic stress had no effect on MeCP2 levels (*p* > 0.05) in comparison with the levels of unstressed animals, in both hippocampus and frontal cortex (Figure 7C,D).

### 2.6. Histological Examination of Hippocampus and Frontal Cortex Tissues

#### 2.6.1. Golgi-Cox

**Figure 8:**
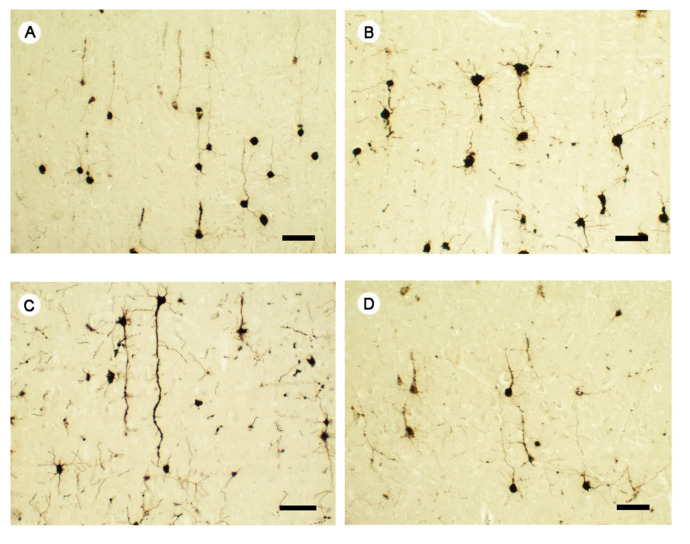
Neuronal morphology after Golgi-Gox impregnation method, in Control and experimental groups. The panel (**A**) showed several pyramidal neurons in the frontal cortex of Control. Stress exposure (**B**) reduced the expansion area of the neurons and determined the increase in thickness of the apical dendritic branches. Escitalopram 5 mg/kg b.w. (**C**) determined a prominent enlargement of the dendritic branches with an increased expansion area compared to Control and stressed animals. However, surprisingly, as with CNP immunoreaction in hippocampus, escitalopram up-titration administration (5 mg/kg then 10 mg/kg b.w.) (**D**) had no effects on the neuronal connections and morphology in the frontal cortex of the treated animals. Each group consisted of 8 rats; ESC5 group received (oral route) escitalopram 5 mg/kg b.w. for 42 days; ESC 5/10 group received (oral route) 5 mg/day b.w. escitalopram for 21 days then 10 mg/kg b.w. escitalopram (oral route) for 21 days; 2′,3′-Cyclic-nucleotide 3′-phosphodiesterase, CNP; A, control; B, stress; C, Stress + ESC5; D, Stress + ESC5/10; ESC, escitalopram. Scale bar = 20 µm.

The stress exposure (Figure 8B) decreased the cortical neuronal extensions and the cells were restricted to soma and short extensions as compared to controls (Figure 8A). The escitalopram administration of 5 mg/kg b.w. improved the cortical neuronal extension, the axons were long and thin and the dendrites expressed many spines as compared to the stressed group (Figure 8C). Escitalopram administration in an up-titration regimen had no effects on the neuronal connections and morphology in the frontal cortex of the treated animals. 

#### 2.6.2. 2′,3′-Cyclic-nucleotide 3′-phosphodiesterase (CNPase)

**Figure 9:**
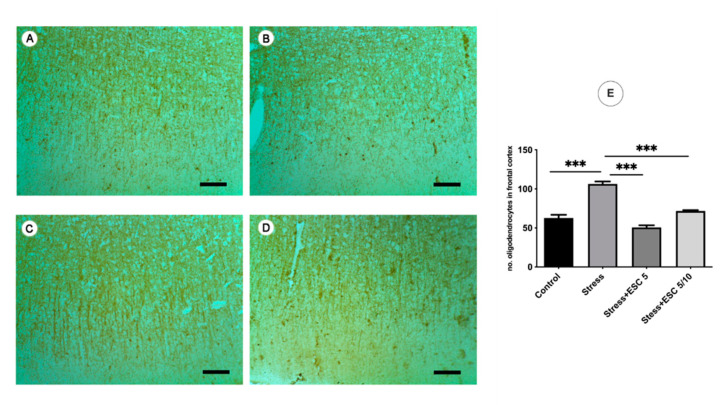
Immunohistochemical labelling of the cortical CNP+ oligodendrocytes in Control and experimental groups. As compared to Control (**A**), the stress exposure (**B**) has induced the significant oligodendrocytes proliferation in the frontal cortex as was showed in (E) (*p* < 0.001). The escitalopram administration in 5 mg/kg b.w. (**C**) and up-titration regiment (5 mg/kg b.w. then 10 mg/kg b.w.) (**D**) normalized the number of the oligodendrocytes as compared to Control without a dose-dependent action (**E**), (*p* < 0.001, *p* < 0.001 respectively). A, control; B, stress; C, Stress + ESC5; D, Stress + ESC5/10; ×200; scale bar = 50 µm; Each group consisted of 8 rats; ESC5 group received (oral route) escitalopram 5 mg/kg b.w. for 42 days; ESC 5/10 group received (oral route) 5 mg/day b.w. escitalopram for 21 days then 10 mg/kg b.w. escitalopram (oral route) for 21 days; Results are expressed as mean ± SD; ANOVA and Tukey’s *post hoc* test; *** *p* < 0.001; ESC, escitalopram; no, number.

**Figure 10:**
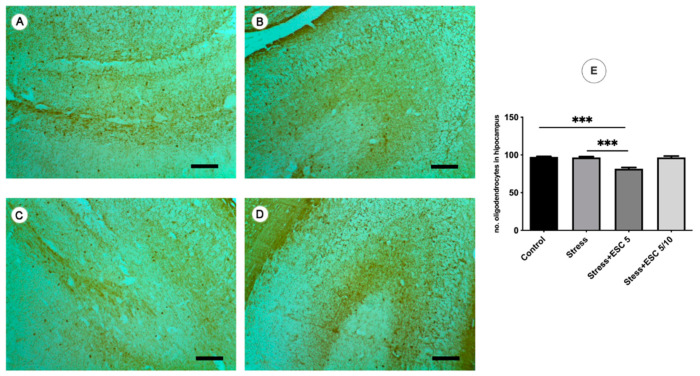
Immunohistochemical detection of the CA3 hippocampal CNP+ oligodendrocytes in Control (**A**) and experimental groups. The stress exposure (**B**) and escitalopram up titration regimen (5 mg/kg b.w. then 10 mg/kg b.w.) (**D**) did not change the number of the CNP+ oligodendrocytes in the CA3 area as compared to Control. Interestingly, escitalopram 5 mg/kg b.w. (**C**) significantly reduced (**E**) the number of the CNP+ oligodendrocytes in the CA3 hippocampus area (*p* < 0.001). A, control; B, stress; C, Stress + ESC5; D, Stress + ESC5/10; ×200; scale bar = 50 µm; each group consisted of 8 rats; ESC5 group received (oral route) escitalopram 5 mg/kg b.w. for 42 days; ESC 5/10 group received (oral route) 5 mg/day b.w. escitalopram for 21 days then 10 mg/kg b.w. escitalopram (oral route) for 21 days. Results are expressed as mean ± SD; ANOVA and Tukey’s *post hoc* test; *** *p* < 0.001; ESC, escitalopram; no, number.

Stress increased the number of oligodendrocytes in the frontal cortex as compared to non-stressed animals (*p* < 0.001) (Figure 9E). Also, escitalopram administered in both regimens (ESC5 and ESC5/10) normalised the number of oligodendrocytes in the frontal cortex as compared to the stressed group (*p* < 0.001) (Figure 9E). In the hippocampus, in the ESC5 group, a smaller number of oligodendrocytes were found compared to the stress group (*p* < 0.001) (Figure 10E).

## 3. Discussion

In the present study, we scrutinised the potential effects of different dose regimens of escitalopram in the treatment of stress-induced depression in rats. More precisely, the results presented here show that chronic stressors determined an increase in the oxidative stress and caspase-3 activity and modulated BDNF and MeCP2 levels in the hippocampus and frontal cortex, along with the occurrence of depressive behaviour. Escitalopram dose increment regimen (5 mg then 10 mg/day b.w.) significantly suppressed the MDA levels, a gauge of membrane lipid peroxidation, in the hippocampus as well as in the frontal cortex. In addition, escitalopram administered in both doses rescued the antioxidant defence by increasing the GSH and GSH/GSSH ratio levels in the hippocampus tissue. But, in the frontal cortex, these changes have been observed only when the escitalopram up-titration regimen was employed. Through all of the above-mentioned effects, escitalopram demonstrated antioxidant proprieties against chronic stress-induced brain oxidative stress. Furthermore, ESC 5/10 reversed the apoptosis activation by lowering caspase-3 activity in the hippocampus. Escitalopram 5/10 showed pro-neurotrophic effects, improved the BDNF expression in the hippocampus and MeCP2 in the hippocampus and frontal lobe in parallel with increasing cortical neuronal extensions. Moreover, escitalopram normalised the number of oligodendrocytes in both analysed tissues.

Stress has the role of a warning sign to threats from the environment and encourages the correction of homeostatic imbalance. If stress is time-limited and appropriate to one’s own capacities, it drives to a motivational state and is a marker of successful functioning and adaptation [38]. On the other hand, repeated stress exposure (i.e., chronic stress) has biological repercussions due to body constant adjustments which can lead, as a result, to stress-related diseases including depression [9]. Stress is incriminated by a large body of evidence in the pathogenesis of depressive disorder. It was found that depressive individuals had an increased level of stressors before the onset of the illness and that stress experienced during childhood was a risk factor for depression in adulthood mainly due to stress-induced epigenetic vulnerability [39,40]. CUMS rat model of depression is a strongly established experimental model for studying the neurobiology of depression and the underlying mechanisms of antidepressant drugs. It consists of the application, over a period of several weeks, of varying mild stressors that resemble the common unpredictable stressors in human daily life and induces an array of behavioural signs that correspond to those seen in depressive patients: anhedonia (i.e., lower preference of sucrose), locomotor inhibition and anxiety like behaviour. Anxiety is frequently associated with depression [41,42,43]. CUMS paradigm was used to assess the possible underlying mechanisms of escitalopram’s antidepressant effects. Escitalopram is an established antidepressant recommended as first line treatment of depression by current international guidelines [44]. Also, several studies proved escitalopram’s antidepressant effect on rat behaviour induced by chronic unpredictable stress, including improvement of the sensitivity to reward (i.e., sucrose preference) or anxiety measured by EPM [45,46,47,48,49,50,51]. In line with previous research, our results demonstrated that escitalopram, either in 5 mg/day b.w. fixed dose or in an up-titration regimen, alleviated depression behaviour by reversing the hedonic deficit induced by chronic stress. Moreover, escitalopram up-titration regimen improved the locomotor activity and also reduced the anxiety, both impelled by chronic stress. Forced swim test is another test designed to assess the signs of depressive-like behaviour, namely the behavioural despair [52]. When placed in a water-filled and unescapable container, the animal is forced to escape but eventually will be immobile. Depressed rodents show greater periods of immobilisation [53]. Several recent papers documented that rats submitted to a chronic stress procedure and then treated with escitalopram showed a decrease of the immobilisation time as compared to untreated rats. In most of the cases, chronic stress determined an increase in the immobility up to 150–200 s, while escitalopram reduced the time to under 100 s [54,55,56,57].

The brain is the main regulator of the stress response via HPA axis which leads to the production of glucocorticoids (cortisol in humans, corticosterone in rodents) upon activation by stressors. Excessive stress and its consequence, increased exposure to glucocorticoids, determine a dysfunctional HPA axis with an abnormal pattern of glucocorticoids secretion and subsequent depression. These effects are brought about by the loss of negative feed-back via downregulation of glucocorticoid receptors in the hippocampus [58,59]. Under stress conditions, it has been shown that corticosterone activates the cytosolic glucocorticoid receptor leading to an increase in the neuron metabolic rate. During the ATP production of neurons, ROS (i.e., superoxide and hydroxyl radical) synthesis occurs as a physiological effect. Instead, ROS levels are thought to increase under chronic stress leading to a state of oxidative stress that in turn will produce protein carbonyl, DNA damage, and peroxidation of the membrane lipids [60]. All these processes can lead in the end to neurons’ apoptosis [17]. Also, the brain is more susceptible to oxidative stress due to the high amount of polyunsaturated fatty acids, high energy needs and low antioxidant reserve [29,61,62]. Several studies support these data and showed that chronic stressed rats had increased reactive oxygen species markers (e.g., thiobarbituric acid reactive substances–TBARS, MDA, protein carbonyl) or lowered antioxidant capacity (e.g., Trolox equivalent antioxidant capacity, GSH, GPx, catalase, peroxiredoxins, superoxide dismutase-SOD) in the whole brain, frontal cortex or hippocampus in comparison with control animals [11,31,54,55,63,64,65,66,67,68,69,70,71]. Other studies have shown that an enhancement of lipid peroxidation (MDA levels) or an increase in antioxidant defence (catalase, GSH, catalase and GPx1 mRNA expressions) failed to appear under CUMS procedure in the rat hippocampus or frontal cortex [19,31,55,68,69]. Our study provides evidence for the involvement of oxidative stress in the depressive-like behaviour in rats exposed to chronic stress. To verify if chronic stress has a biological resonance regarding oxidative stress, we quantified the levels of MDA and demonstrated that chronic stress enhanced lipid peroxidation in depressive animals. GSH and GSH/GSSG levels in the hippocampus and frontal cortex were depleted in comparison with non-stressed animals. Also, catalase activity showed a trend to increase in both examined brain areas after chronic stress and this may be in response to the intensification of oxidative stress.

Escitalopram operates through a selective serotonin reuptake inhibition mechanism; therefore, it leads to an increase of serotonin in the synaptic cleft and an improvement of the functionality of synapses [72]. It is not sure whether this represents the only mechanism implicated in the escitalopram antidepressant role. Treatment with different selective serotonin reuptake inhibitors, including escitalopram, was proven to downregulate oxidative stress. However, current research provides limited evidence regarding the effect of escitalopram on oxidative stress markers in different brain areas of stress-depressed rats. Escitalopram was demonstrated to regulate CUMS induced oxidative stress by suppressing lipid peroxidation (i.e., MDA or TBARS) or increasing the levels of antioxidant molecules (i.e., GSH, GPx, catalase, SOD) in the whole brain or prefrontal cortex [31,49,54,64,65,67]. Conversely, Eren et al. (2007) and Martin-Hernandez et al. (2018) reported decreased lipid peroxidation (quantified by MDA or TBARS) in the prefrontal cortex of CUMS subjected rats, while Wigner et al. (2020) showed that catalase mRNA expression in the hippocampus and cerebral cortex was reduced after escitalopram administration in comparison with stressed animals [19,31,64]. Our results are partially consistent with these data. We showed that both escitalopram treatment regimens decreased MDA levels in the hippocampus but not in the frontal cortex. Also, the non-enzymatic antioxidant defence, namely GSH and GSH/GSSG ratio, was replenished by escitalopram up-titration regimen in the hippocampus and frontal cortex. Also, in our study, escitalopram therapy had a tendency to normalise catalase activity. Moreover, studies have outlined that GSH is the major cerebral antioxidant while catalase is contained in the brain in small amounts [73,74]. Based on these data, we could hypothesize that escitalopram targets depression associated oxidative stress through GSH/GSSG redox cycle, therefore this could lead to normalisation of catalase in response to the reduced oxidative stress. It was demonstrated that a deplete of serotonin (5-hydroxytryptamine, 5-HT) in the brain is accompanied by a decrease of antioxidants levels and an increase of oxidative stress markers. Moreover, exogenous administration of 5-hydroxitryptophan (5-HTP), a precursor of 5-HT, prevented these effects, therefore suggesting an anti-oxidative protective effect of 5-HT and 5-HTP [75,76]. The hydroxylated phenolic ring present in the chemical structure of these molecules was suggested to underlie this effect [75,77,78]. Also, 5-HT was revealed to bind to lipid membranes and act as a scavenger of the reactive oxygen species in order to prevent the peroxidation [78,79]. As far as the current literature goes, escitalopram’s anti-oxidative effects may rely on increasing serotonin availability via inhibition of serotonin reuptake, but further studies that tackle other potential mechanisms are needed.

The hippocampus and frontal cortex play a key part in the pathophysiology of depression through their roles in executive and cognitive functions, and emotions regulation [80]. Both the hippocampus and the frontal cortex are sensitive to stress, a major contributor to the onset of depression [81,82]. For instance, patients with depression showed hippocampus and frontal cortex atrophy [83,84]. Correspondingly, in rat model of stress induced depression, the hippocampus and prefrontal cortex had reduced volumes [85,86]. Moreover, chronic antidepressant therapy mitigates these volumetric changes that have been suggested to appear due to increased apoptosis or impaired neurogenesis [14,87]. Since BDNF is involved in neuronal differentiation, survival and development, while antidepressant therapy upregulates its levels, it is suggested to play a part in these changes along with increased apoptosis and oxidative stress [14]. Research indicates that rats subjected to chronic stress have increased capsase-3, a pivotal enzyme to induce apoptosis, in the brain cortex, frontal cortex or hippocampus [18,88,89,90,91]. We report similar results. Moreover, treatment with fluoxetine, an antidepressant drug with a similar pharmacological mechanism to escitalopram, was shown to bring to normal caspase-3 or Bcl-2 activity in chronic stress rat model [92]. Evidence regarding escitalopram effects on caspase-3 is limited. In a chronic unpredictable mild stress rat model of depression, Yang et al. (2018) described a decrease of caspase-12 after escitalopram treatment [93]. Capsase-12 serves as a signalling molecule for activation of caspase-3. In our study, escitalopram administered in the up-titration regimen (5 mg then 10 mg/kg b.w.) determined a decrease of caspase-3 activity in the hippocampus but no effect was observed in the frontal cortex. Although speculative, we could suggest that escitalopram has a dose-dependent anti-apoptotic activity which needs further investigation to advance our current understanding.

After gene expression, BDNF is translated into pro-BDNF and then cleaved into the mature form. Mature BDNF binds to tyrosine kinase B receptor (TrkB) and consequently leads to the activation of several signalling pathways [94]. Dendritic atrophy and neuronal cell death have been tied to BDNF decreased levels observed in response to chronic stressors. Different antidepressants reversed these changes coupled with ameliorations of depressive-like behaviour [95,96]. Chronic stress was reported to determine a reduction in BDNF mRNA or protein content in the rat hippocampus or frontal cortex [35,95,97,98,99]. But these data should be interpreted with caution since results at variance with are found in the literature. BDNF protein or mRNA levels were shown to be unchanged or even increased in the hippocampus, frontal cortex or whole cortex of stress-induced depressed rats [96,100,101,102]. This could be explained by a reduced turnover of BDNF, therefore a higher level in the tissue that is not released [103]. Also, activated TrkB receptors determine the phosphorylation of an important transcriptional factor, namely cAMP response element binding protein (CREB). Once phosphorylated, CREB promotes the transcription of BDNF gene [94]. It has been demonstrated that increased oxidative stress inhibits the phosphorylation of CREB in the hippocampus and, subsequently, BDNF synthesis [104]. Therefore, another explanation of the BDNF decreased levels in the hippocampus, observed in depressive rats and also in our study, could be the increased oxidative stress. Our results add additional support to existing knowledge regarding a decrease in the BDNF levels in hippocampus when chronic stress was employed. But, in the frontal cortex, stress determined an increase of BDNF, which could suggest a dual effect of stress depending on the cerebral region analysed. Also, the increase of BDNF seen in the frontal lobe might occur due to a possible compensatory mechanism.

A large body of evidence points out to a possible role of BDNF in mitigating antidepressants effect [105]. Regarding clinical reports, several studies documented increased BDNF serum levels in patients treated with escitalopram in comparison with baseline levels [106,107,108]. Lee et al. also observed that non-remitters who underwent escitalopram therapy had lower BDNF post-treatment levels. This data suggests the potential role of BDNF as a marker of clinical response to escitalopram [109]. Chronic treatment with different antidepressants seemed to prevent the upregulation or downregulation of BDNF in the hippocampus or prefrontal cortex in response to stress or even to increase BDNF in comparison with controls [14,96]. Until now, research concerning how escitalopram affects BDNF levels in stress-depressed rats is rather ambiguous. On the one hand, treatment with escitalopram did not show any effect regarding BDNF in prefrontal cortex or hippocampus of stressed rats [56,102]. On the other hand, several studies carried out using stress paradigm in rats revealed that escitalopram successfully rescued BDNF protein or mRNA levels in the hippocampus [51,57]. These results are not inconsistent with our findings that both fixed or up-titrated regimens of escitalopram up-regulated BDNF protein levels in the hippocampus of CUMS rats. Conversely, we report that escitalopram 5 mg/kg b.w. had no effect on the BDNF levels in the frontal cortex while ESC 5/10 restored the levels of this neurotrophin to healthy group levels. In an attempt to integrate our results regarding the effect of stress and the role of escitalopram, we could speculate that escitalopram regulates BDNF depending on the consequence of stress in each brain areas. As mentioned before, BDNF is suggested to reduce the oxidative stress damage via Nrf2 pathway, leading in the end to upregulation of antioxidant molecules [11,28]. The enhanced antioxidant defence seen in the hippocampus of the groups treated with escitalopram could therefore be explained by the elevated BDNF levels noticed in the same groups.

In the recent years, growing evidence supports the involvement of Mecp2 in the pathomechanism of depression and antidepressants’ action. MeCP2 is involved in the regulation of BDNF but the dynamics of this interaction is still controversial. Some studies incriminate MeCP2 as a repressor of BDNF transcription while other are in favour of an activation role. However, a dual operating model has been proposed, since neuronal-activity-induced phosphorylation of MeCP2 determined the recruitment of regulatory complexes that activate or repress BNDF transcription [34]. In addition, MeCP2 deficiency is associated with abnormally derepressed BDNF silencers which are suggested to explain the BDNF default seen in Rett syndrome [110]. Su et al. (2015) observed that MeCP2 knockdown hippocampal neurons had reduced levels of BDNF, whereas BDNF and MeCP2 protein levels were decreased in chronic stressed rats with depressive-like symptoms [35]. Also, caregiver maltreatment favoured decreased MeCP2 mRNA levels in the medial prefrontal cortex of adolescent and adult male rats [111]. Moreover, McGill et al. (2006) found enhanced anxiety and increased stress-induced corticosterone in MeCP2 deficient mice [112]. These results attest the implication of MeCP2 in the stress-induced disorders. In addition, it is suggested that one of the action pathways of antidepressant drugs requires MeCP2. More precisely, several antidepressants (e.g., escitalopram) were demonstrated to phosphorylate MeCP2 in order to increase BDNF expression [36,113,114,115]. Our results are in partial agreement with previous studies. We report no effect of chronic stress on the protein levels of MeCP2 in the hippocampus and frontal cortex. Instead, our study demonstrated an increase in MeCP2 protein levels in both examined brain areas following ESC5/10 treatment. Also, escitalopram seems to modulate MeCP2 protein levels in a dose-related manner. Further studies are needed in order to shed more light on the current understanding of the relationship between MeCP2 levels, depression and escitalopram treatment.

Depression is associated with axonal loss and impaired oligodendrocytes could contribute to this result since they have an important role in myelin generation and axon integrity [116,117]. The myelin sheath is implicated in the neuronal transmission and the metabolic and trophic support of the sheathed neurons [117]. Chronic unpredictable stress-induced depression was demonstrated to decrease the CNP+ cells in some regions of the hippocampus (e.g., CA1 or CA3 field, dentate gyrus) or in the medial prefrontal cortex of rats [118,119,120]. Also, different antidepressant therapies (i.e., desvenlafaxine treatment or physical exercise) normalised the oligodendrocytes’ marker CNPase in stress-depressed rats [120,121]. Fluoxetine reversed the reduction of the number of oligodendrocytes determined by chronic stress [119]. In contrast, restrain induced stress or corticosterone administration promoted oligodendrogenesis in rat hippocampus [122]. Kaul et al. suggested this might probably be a compensatory consequence due to the loss of oligodendrocytes’ roles [123]. Our results are in partial agreement with those from current literature. Our study showed that chronic stress up-regulated the number of oligodendrocytes in the frontal cortex but had no effect in the hippocampus. Also, the number of oligodendrocytes was brought to normal by escitalopram dose-increment regimen in the frontal cortex. However, the possible compensatory up-regulation effect of oligodendrocytes entailed by chronic stress needs further investigation to unfold the precise underlying mechanism.

Several lines of evidence point out that hippocampus is the most susceptible to oxidative stress determined either by psychological or pharmacological means. Frontal cortex seems to be more resilient to oxidative stress [124,125]. These results are partially in agreement with ours, including apoptosis. Moreover, oxidative and nitrosative stress deplete the 5-HT (5-hydroxytryptamine) content via oxidation of tetrahydrobiopterin, an essential cofactor for the synthesis of monoamine neurotransmitters [126]. As abovementioned, oxidative stress was documented to inhibit the phosphorylation of CREB, a major promotor of BDNF gene transcription. As a result of this action, oxidative stress inhibits the BDNF synthesis [94,104,127]. Also, low BDNF was documented to prevent the translocation of Nrf-2 and therefore to lower the antioxidant defence leading to pro-oxidative state [11,28]. Also, serotonin and BDNF have a dual interaction, enhancing each other synthesis [128]. We suggest that BDNF decreased levels in the hippocampus could be explained by the increased oxidative stress damage and possible subsequent 5-HT decrease. On the other hand, frontal cortex, due to its oxidative stress resilience and consequently unaffected BDNF levels, could even increase BDNF in a protective way. Escitalopram determines an increase in the extracellular 5-HT as a result of its pharmacological properties. Therefore, we could hypothesize that escitalopram restores stress-induced alterations in a brain region dependent mode via this mechanism. All these possible mechanisms deserve further research.

In addition, the histological findings of the present study advocate for the escitalopram rescuing role regarding neuroplasticity and trophicity. More precisely, stress induced improper trophic support probably coupled with increased oxidative stress led to decreased cortical neuronal extensions and the cells were restricted to soma and short extensions. Escitalopram increased the cortical neuronal extension, the length of the axons and the spines expressed by dendrites.

## 4. Materials and Methods

### 4.1. Reagents

Escitalopram oxalate, *o*-phthalaldehyde, reduced glutathione and Bradford reagent were from Sigma-Aldrich Chemicals GmbH (Munich, Germany), while EDTA-Na_2_ and 2-thiobarbituric acid were purchased from Merck KGaA (Darmstadt, Germany). Absolute ethanol, hydrogen peroxide and n-butanol were obtained from Chimopar (Bucharest, Romania). Antibodies against BDNF, MeCP2, β actin and secondary peroxidase-linked antibodies were from Santa Cruz Biotechnology (Santa Cruz, CA, USA), while phosphorylated histone. ELISA tests for caspase-3 were purchased from Elabscience (Houston, TX, USA) and Bradford total protein concentration assay was from BioRad (Hercules, CA, USA).

### 4.2. Animals and Housing

The experiments were performed on 32 male Wistar rats (180 ± 20 g). The animals were acclimatised to the laboratory conditions (12 h light/dark schedule, room temperature 24 ± 2 °C) for 7 days before the experiments began. During acclimatisation, rats had free access to standard normocaloric pellet food and water was provided *ad libitum*. The experiment was performed in the Laboratory for Experimental Research, Physiology Department, ‘Iuliu Hatieganu’ University of Medicine and Pharmacy, Cluj-Napoca, Romania. Prior approval of the experimental procedures was obtained from Animal Research Ethics Board of “Iuliu Hatieganu” University of Medicine and Pharmacy and from the Department for Veterinary Surveillance and Food Safety, Cluj-Napoca branch, according to the Directive 2010/63/EU on the protection of animals used for scientific purposes (approval No. 169/06.06.2019).

### 4.3. Experimental Design

**Figure 11:**
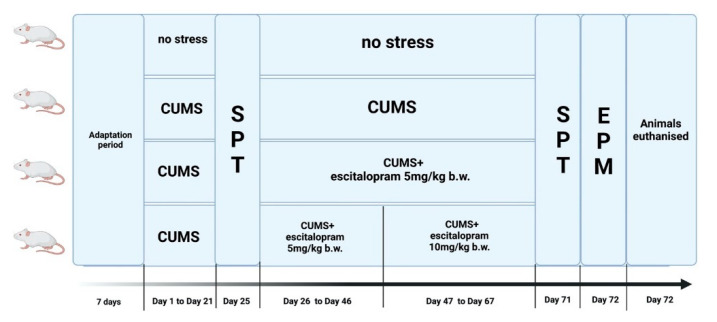
Illustration of the experimental design. Each group consisted of 8 rats and escitalopram was administered by gavage (oral route); CUMS, chronic unpredictable mild stress; SPT, sucrose preference test; EPM, elevated plus maze; Figure 11 was created using BioRender software (accessed on 15 June 2021).

After the acclimatisation period, the rats were randomly divided into 4 groups (n = 8) and treated as follows: (1) control: unstressed + vehicle; (2) stress: CUMS + vehicle; (3) stress + ESC5: CUMS + escitalopram 5 mg/kg b.w.; (4) stress + ESC5/10: CUMS + escitalopram 5 mg/kg b.w. from day 26 to day 46 and then CUMS + escitalopram 10 mg/kg b.w. from day 47 to day 67. Vehicle or escitalopram were administered by oral route (gavage) from day 26 to day 67 of the experiment. The antidepressant treatment was given after onset of depression in order to reflect the real clinical setting. The escitalopram dose was increased in group 4 in order to mimic the clinical situation where usually a patient receives different doses of antidepressant [129]. The doses of escitalopram were chosen based on previous research [130]. Animals from the control and CUMS group received an equivalent dose of carboxymethyl cellulose 2%. On day 25 and on day 71 the SPT was performed. EPM was accomplished on day 72. In the last day of the experiment, animals were sacrificed under general anaesthesia performed with a 90 mg/kg b.w. ketamine, 10 mg/kg b.w. xylazine cocktail. Hippocampus and frontal cortex were isolated and the tissues were divided in half. One half of the tissues were used for the biochemical analysis while the other half for histological and immunohistochemical investigation. The experimental design is illustrated in Figure 11.

### 4.4. Chronic Unpredictable Mild Stress Protocol

The CUMS protocol in this study consisted in mild stressors as described previously but slightly modified [131]. The CUMS paradigm lasted for 63 days. Rats from the control group were housed individually in a separate room and had free access to food and water. Animals from stress, stress + ESC5 and stress + ESC5/10 groups were separated in individual cages and were subjected daily to a different stressor as follows: (1) 24 h of food and water deprivation, (2) 17 h of cage tilting at 45°, (3) 17 h period of soiled cage (200 mL of water in the sawdust bedding), (4) forced swimming for 5 min, (5) tail pinching for 5 min, (6) 17 h of clean cage without bedding, (7) 12 h dark cycle replaced by continuous light. One stressor was applied each day and the scheduled was designed to be unpredictable.

### 4.5. Behavioural Testing

#### 4.5.1. Sucrose Preference Test

The sucrose preference test was used in order to observe anhedonia (described as a decreased ability to experience pleasure) and was performed as previously reported with minor modifications [10,54,132,133]. The SPT was conducted over a 24 h period of time, before escitalopram administration started, on day 25 and at the end of the experiment, on day 71. Prior to the test, the animals were trained to consume a 2% sucrose solution. The training phase consisted of a 48-h period of time where the animals were given two bottles containing 2% sucrose solution. After a 12 h period of food and water deprivation, the animals were given access for 24 h to two bottles, one containing 2% sucrose solution and one containing tap water. The positions of the bottles were switched after 12 h to prevent side preferences. At the end of the test, the bottles were measured and the volume intakes of sucrose solution and water were determined. Sucrose preference was calculated using the following formula: 

sucrose preference = (consumed sucrose solution/(consumed sucrose solution + consumed tap water)) × 100.

A sucrose preference below 65% is usually considered a hallmark of anhedonia, considering that control animals have ≥65% sucrose preference [134].

#### 4.5.2. Elevated Plus Maze

Elevated Plus-Maze is a behavioural test, widely employed to assess anxiety-like behaviours in rodents or considered the first-choice test for screening anxiolytic drugs [135,136,137]. EPM was conducted as described previously [138]. The EPM test was performed on day 72.

### 4.6. Oxidative Stress Markers

For the evaluation of the oxidative stress, we measured malondialdehyde as a marker of lipid peroxidation and GSH, GSH/GSSG ratio and catalase as parameters of antioxidant activity both in the hippocampus and frontal cortex. Malondialdehyde (MDA) was determined using the fluorimetric method with 2-thiobarbituric acid described by Conti et al. (1991) and the levels were expressed as nmoles/mg protein [139]. Glutathione reduced (GSH) and oxidised (GSSG) were quantified in the tissue homogenates by fluorimetry using *o*-phthalaldehyde [140]. Their concentrations were determined by using standard curves and the results were expressed as nmol/mL or as GSH/GSSG ratios. Catalase was determined as described by Pippenger et al. and results were expressed as U/mg protein [141].

### 4.7. Quantitative Estimation of BDNF and MeCP2 Expressions

Lysates (20 µg protein/lane) were separated by electrophoresis on SDS PAGE gels and transferred to polyvinylidene difluoride membranes, using a BioRad Miniprotean system. Blots were blocked and then incubated with antibodies against: BDNF, MeCP2 and β actin, then further washed and incubated with corresponding secondary peroxidase-linked antibodies. Proteins were detected using Supersignal West Femto Chemiluminiscent substrate and a Gel Doc Imaging system equipped with a XRS camera and Quantity One analysis software (BioRad). β actin was used as a protein loading control.

### 4.8. Histological and Immunohistochemical Investigation of Hippocampus and Frontal Lobe

For the evaluation of histological and immunohistochemical features, the brain tissue was isolated and fixed in 10% neutral formalin solution for 48 h for immunohistochemistry and for 3 weeks in Golgi-Cox solution (5% potassium dichromate, 5% potassium chromate, 5% mercuric chloride).

#### 4.8.1. Immunohistochemistry

After paraffin embedding, sections were cut at 5 µm and mounted on electrostatically charged glass slides. Tissue sections were dewaxed in xylene, rehydrated and the sections were subjected to antigen retrieval with citrate buffer pH 6 at 95 °C for 10 min. and then the slides were treated with 3% hydrogen peroxide for 10 min. After washing with PBS 0.01 M pH 7.4, nonspecific background staining was blocked with 10% BSA in PBS pH 7.4 for 1 h. The sections were then incubated at 4 °C for 12 h with mouse monoclonal anti-rat 2′,3′-Cyclic-nucleotide 3′-phosphodiesterase (CNPase) antibody (AB9342, Sigma-Aldrich Chemicals GmbH), dilution 1:350, and mouse monoclonal anti-rat PSD95 antibody (MABN1120, Sigma-Aldrich Chemicals GmbH) dilution 1:400. Then the sections were washed with PBS and treated with biotinylated-HRP link universal (biotinylated anti-rabbit, anti-mouse, and anti-goat immunoglobulins in PBS (Dako North America Inc., Carpinteria, CA, USA, LSAB+ System-HRP) at room temperature for 15 min. and after PBS washing, incubated with streptavidin-peroxidase (Dako North America Inc., Carpinteria, CA, USA, LSAB + System-HRP) for 15 min. The slides were washed with PBS and incubated with DAB (DAB + Chromogen, Dako North America Inc., Carpinteria, CA, USA, LSAB+ System-HRP) for 5 min. After 3 shorter baths of absolute ethanol and 1 bath of xylene, the sections were covered with synthetic resin (Merck).

#### 4.8.2. Golgi-Cox Impregnation

After 3 weeks of Golgi-Cox impregnation, the coronal slices of the whole brain were subjected to paraffin embedding with the same protocol as for immunohistochemistry. Sections at 25 µm thickness were mounted on gelatine-coated slides and dried for 3 days at 37 °C. The sections were dewaxed in xylene, rehydrated and the slides were treated with 15% NH_3_ solution for 8 min, and then, after washing with tap water, the sections were treated for 5 min with 5% sodium thiosulphate aqueous solution. After 3 × 5 min baths of absolute ethanol and 1 × 5 min bath of xylene, the sections were covered with synthetic resin (Merck).

### 4.9. Statistical Analysis

All statistical analyses were performed using GraphPad Prism software, version 8.0 for Windows [142]. Data are presented as mean ± standard deviation (SD). The comparisons of the results between groups were made by one-way ANOVA analysis of variance, followed by Tukey’s *post hoc* test. For the EPM results, Kruskal-Wallis test was employed followed by Dunn’s multiple comparison test. A *p* value lower that 0.05 was considered to be statistically significant.

## 5. Conclusions

In the present study we bring important data regarding the potential mechanisms that underlie the complex interaction of stress and depression and the antidepressant effect of escitalopram. Our study showed that escitalopram suppressed the effects of increased oxidative stress and enhanced the antioxidant protection in the hippocampus and frontal cortex, along with alleviating stress-induced depressive and anxious behaviours. Moreover, escitalopram proved to have anti-apoptotic and pro-trophic effects by decreasing caspase-3, up-regulating MeCP2 and regulating BDNF, depending on the brain area. Escitalopram also seemed to have a modulating action on the oligodendrocytes’ number. All these effects were mirrored by the ameliorating effect of escitalopram regarding the histological image of neurons, suggesting a protective role of escitalopram against depression associated-neuronal damage. Further studies are necessary to thoroughly detail the interaction of stress, depression and the accordingly cellular and molecular neuronal changes and, also, to better characterise the effects of escitalopram on these potential pathways. These data could help the understanding of the pathogenesis of depression and could lead to the discovery of novel therapeutic targets for antidepressants.

## Data Availability

The data presented in this study are available on request from the corresponding author.
